# Supplementary figures and images for: The PIP4K2 inhibitor THZ-P1-2 exhibits antileukemia activity by disruption of mitochondrial homeostasis and autophagy
Source: Blood Cancer J. 2022 Nov 9;12(11):151. doi: 10.1038/s41408-022-00747-w (PMC9643393; doi:10.1038/s41408-022-00747-w)

# A

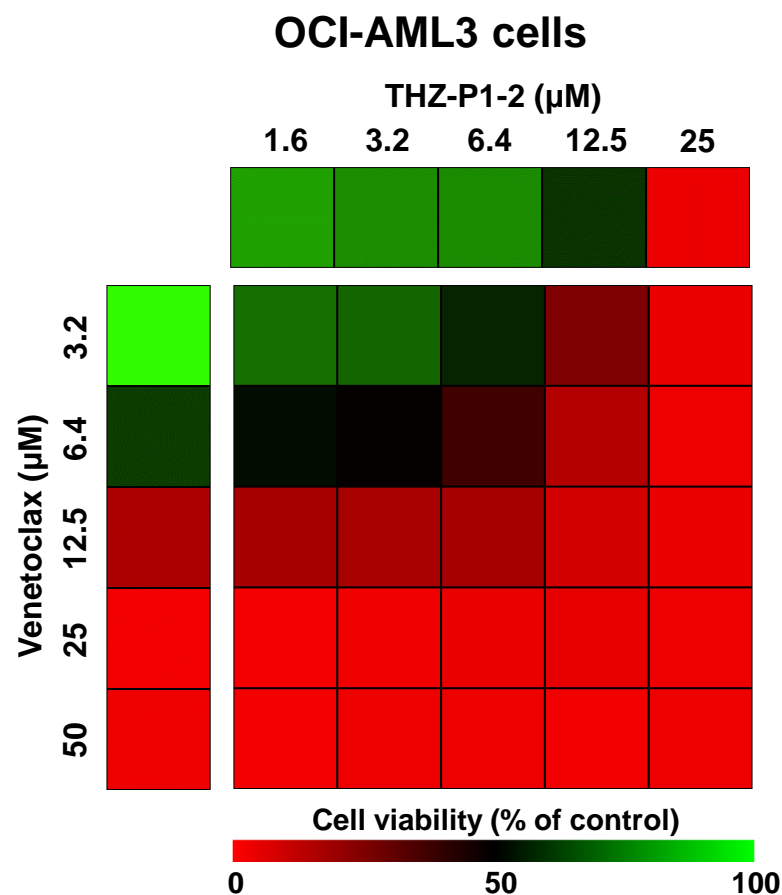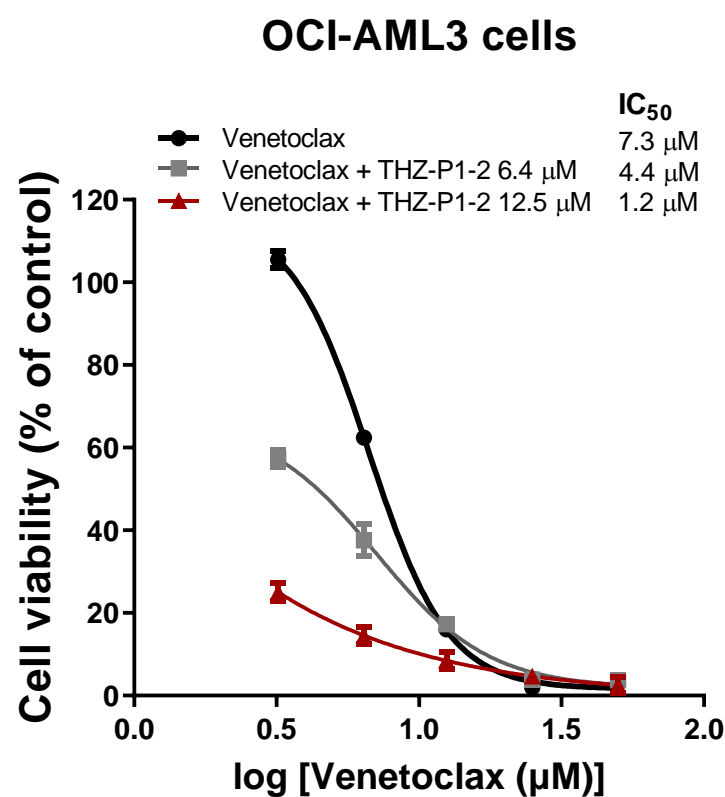

# B

**OCI-AML3 cells**

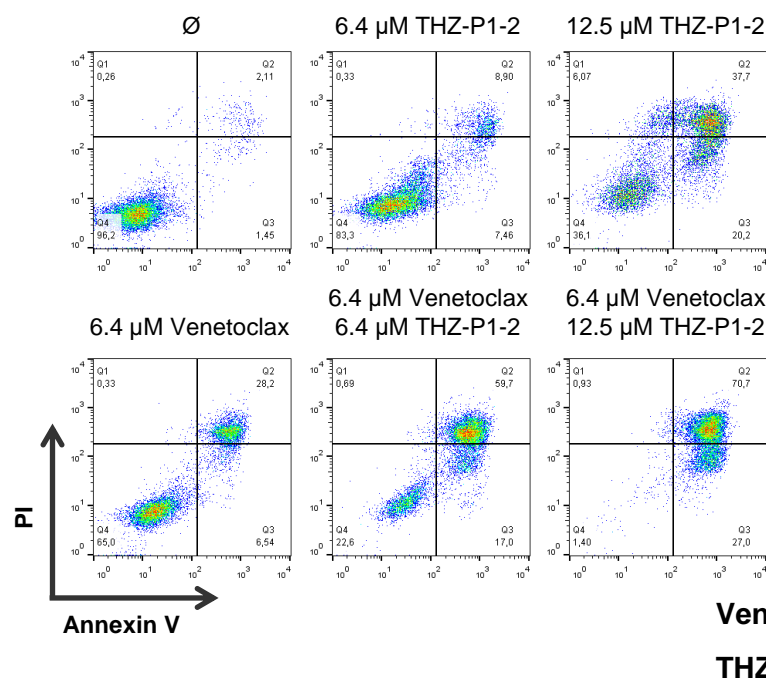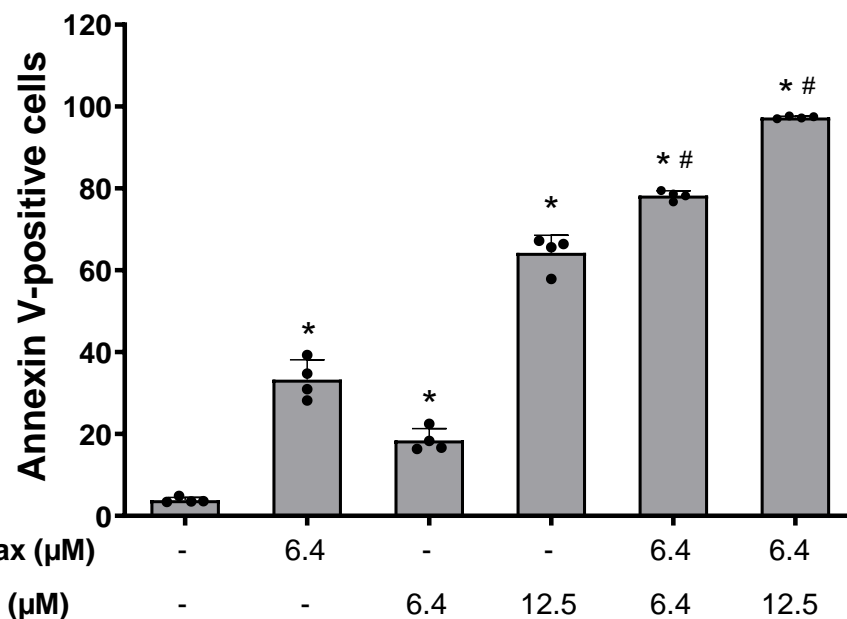

Supplement: Supplementary file 1 — Supplementary Figure 1 [file 41408_2022_747_MOESM1_ESM.pdf]

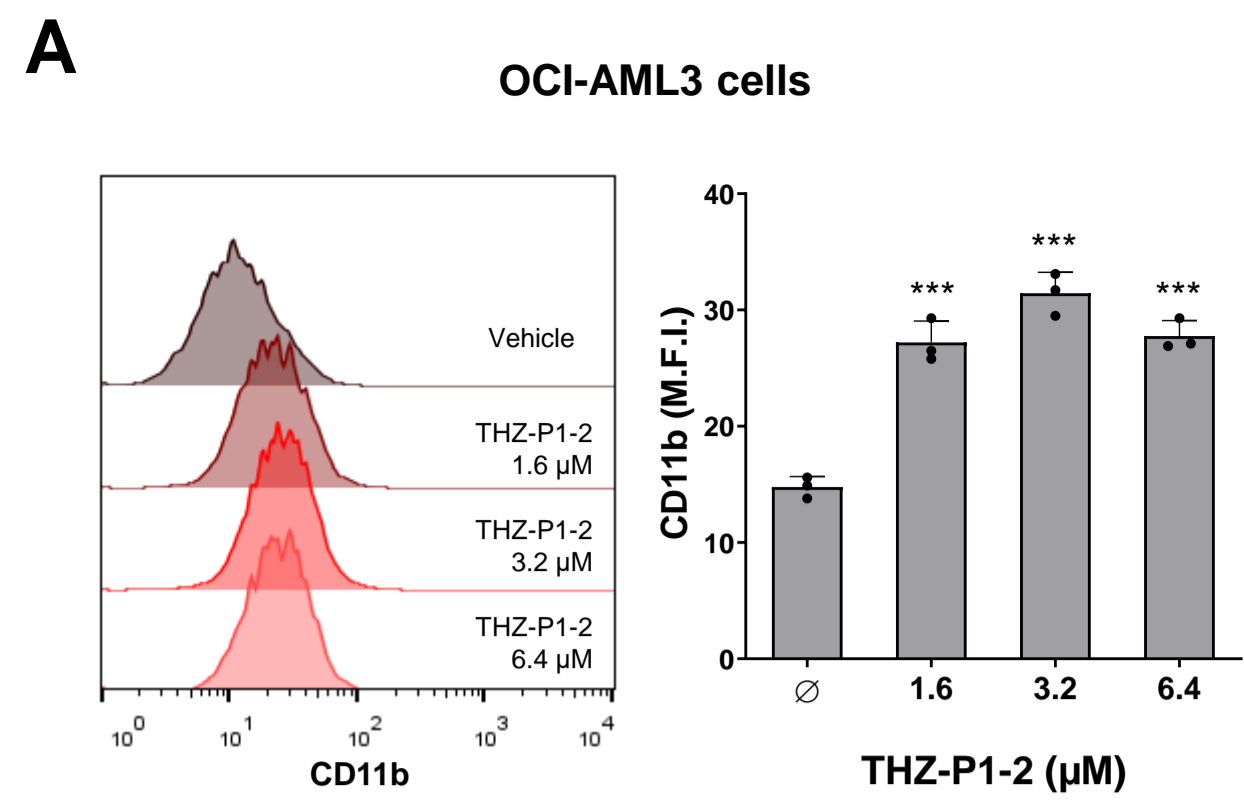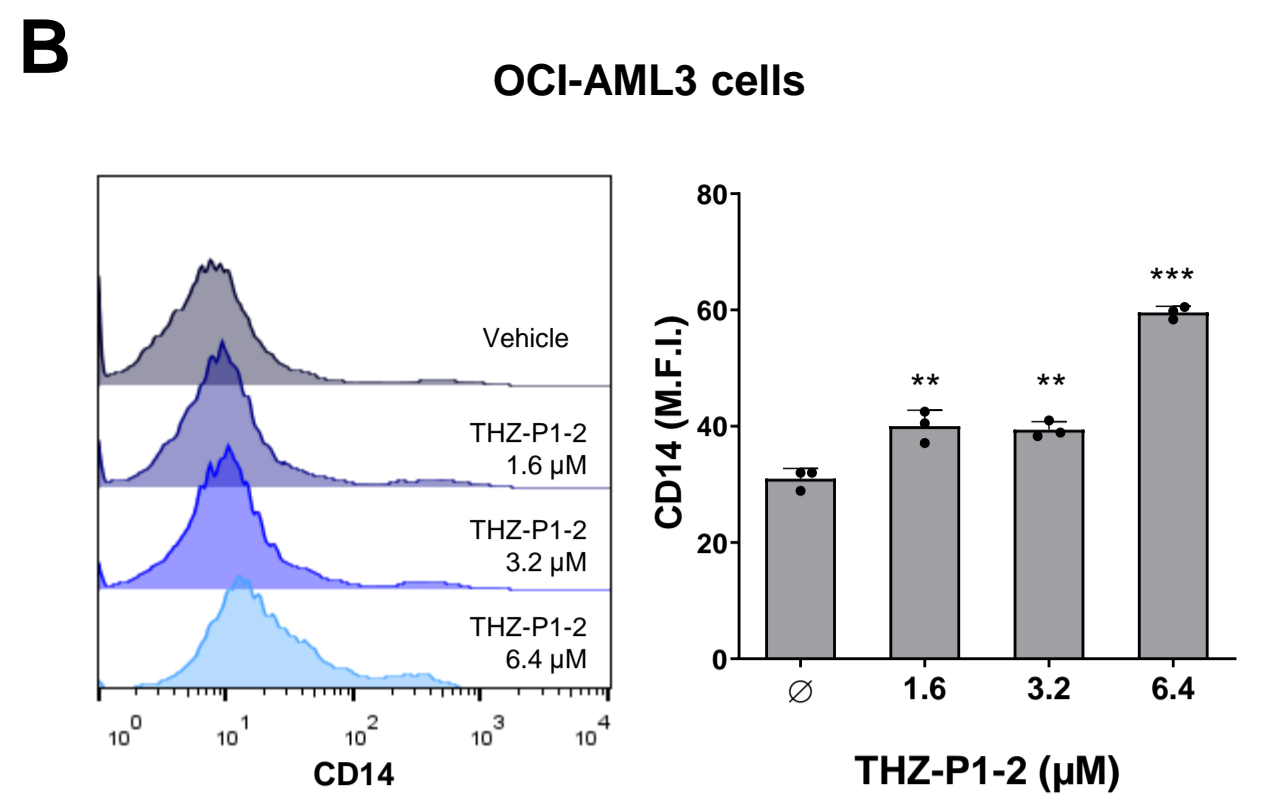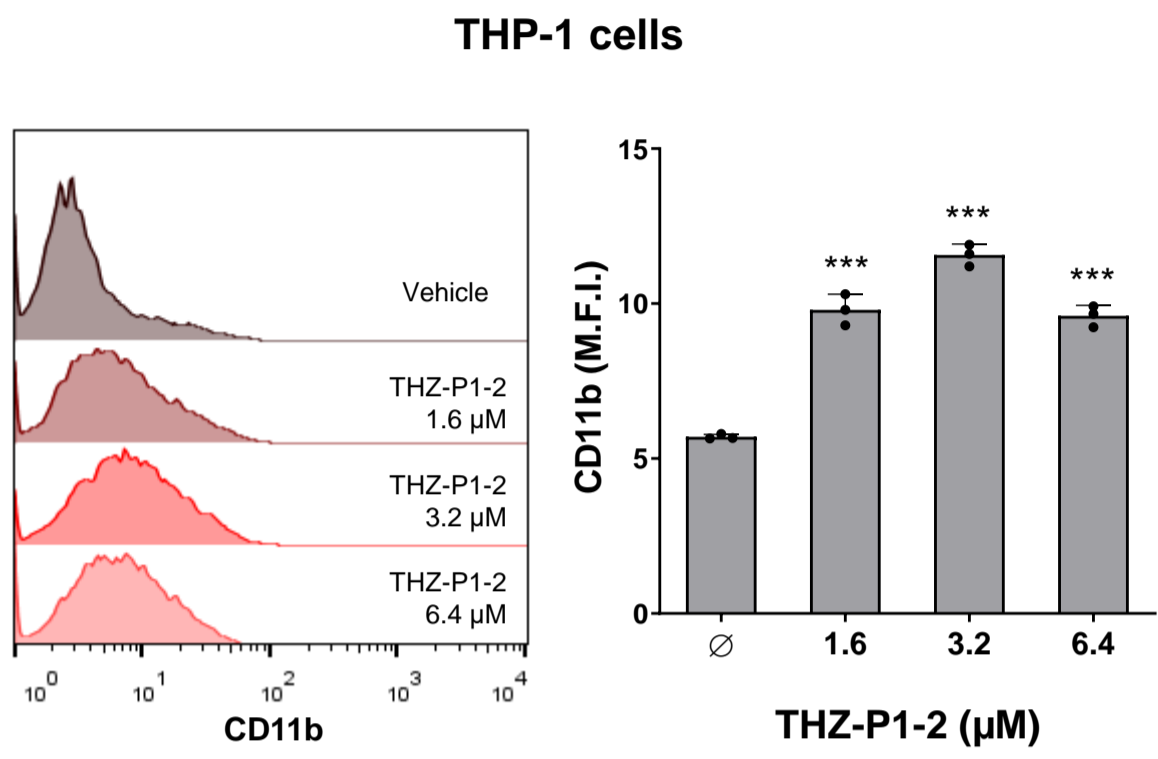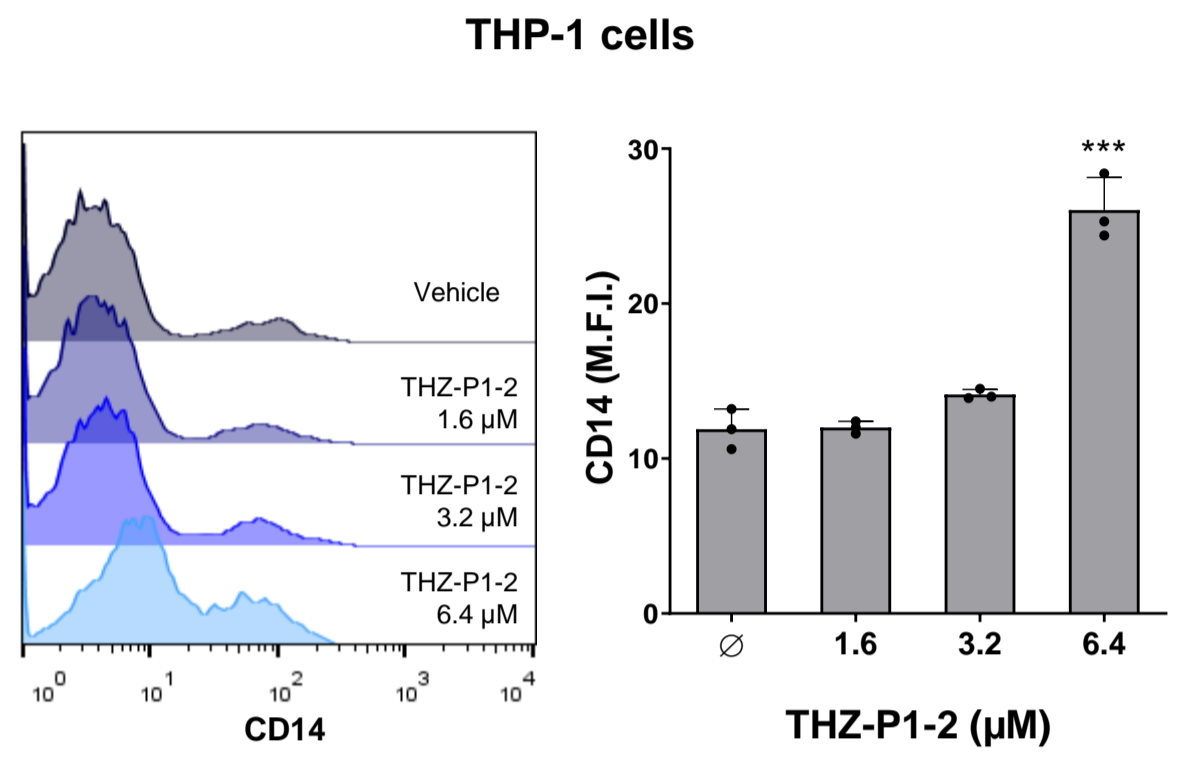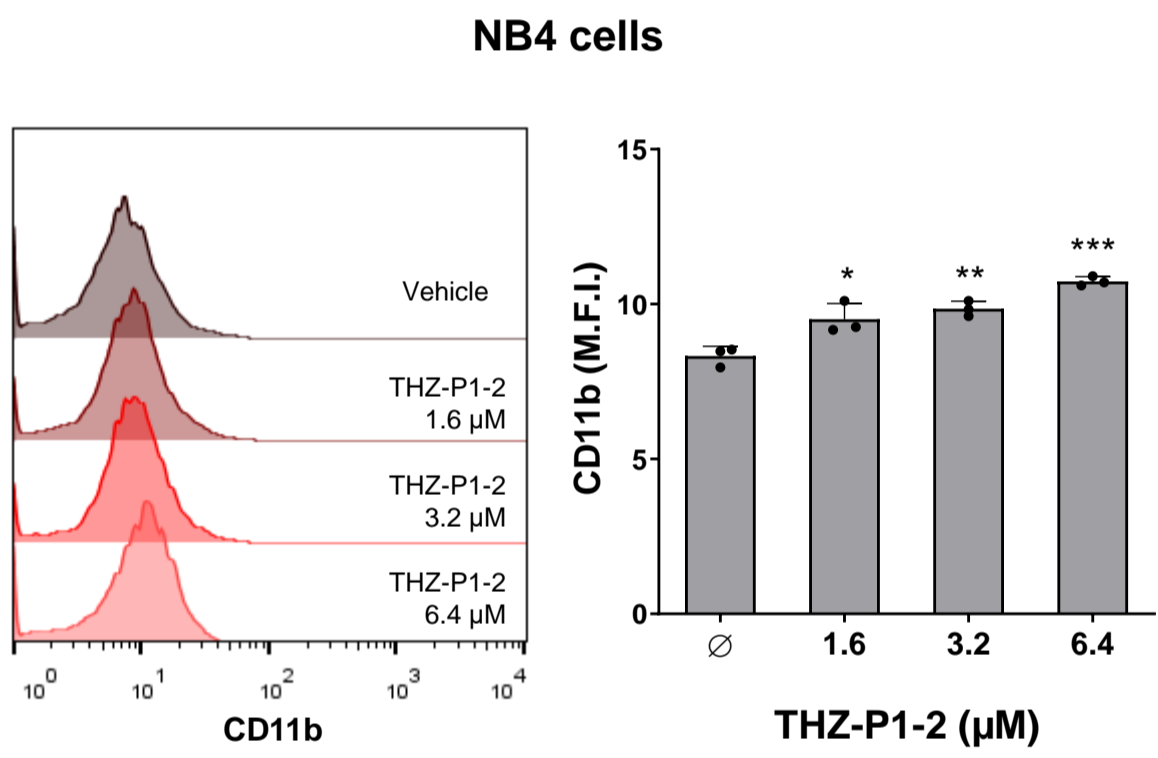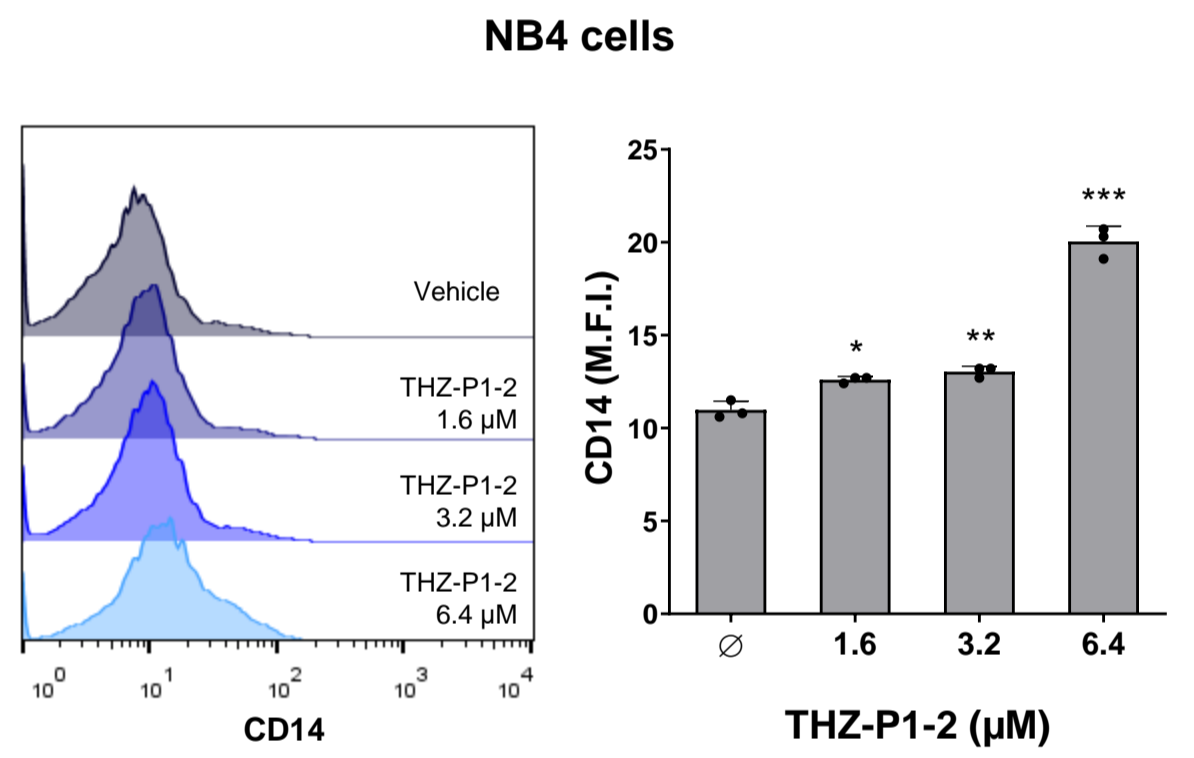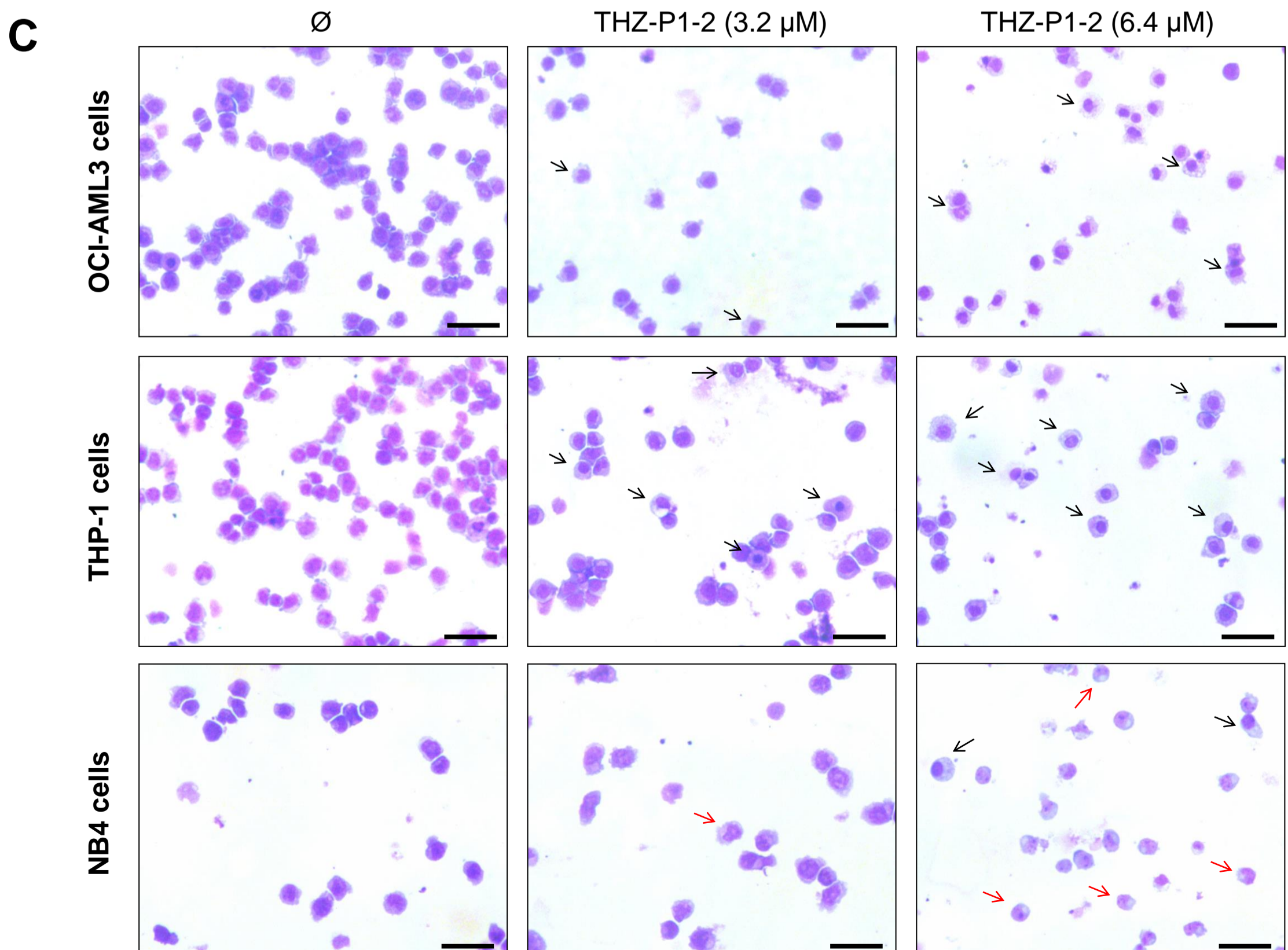

Supplement: Supplementary file 2 — Supplementary Figure 2 [file 41408_2022_747_MOESM2_ESM.pdf]
