# Supplementary material for: The PIP4K2 inhibitor THZ-P1-2 exhibits antileukemia activity by disruption of mitochondrial homeostasis and autophagy
Source: Blood Cancer J. 2022 Nov 9;12(11):151. doi: 10.1038/s41408-022-00747-w (PMC9643393; doi:10.1038/s41408-022-00747-w)
Supplement: Supplementary file 3 — Supplementary Figure Legends [file 41408_2022_747_MOESM3_ESM.doc]

**Figure legends**

**Supplementary Figure 1. THZ-P1-2 potentiates venetoclax-induced apoptosis in OCI-AML3 cells. (A)** Dose-response cytotoxicity for combined treatment was analyzed by methylthiazoletetrazolium (MTT) assay for OCI-AML3 cells treated with graded concentrations of venetoclax and THZ-P1-2 alone or in combination with each other for 48 h, as indicated. Values are expressed as the percentage of viable cells for each condition relative to vehicle-treated cells. Results are shown as the mean of at least three independent experiments. Note that the inhibitory concentration of 50% (IC_50_) for venetoclax was reduced in combination with THZ-P1-2 in OCI-AML3 cells. **(B)** Apoptosis was detected by flow cytometry in OCI-AML3 cells treated with venetoclax and/or THZ-P1-2 for 48 hours using an APC-annexin V/PI staining method. Representative dot plots are shown for each condition; the upper and lower right quadrants (Q2 plus Q3) cumulatively contain the apoptotic population (annexin V^+^ cells). Bar graphs represent the mean ± SD of at least three independent experiments. The p values and cell lines are indicated in the graphs; **p* < 0.05 for venetoclax- and/or THZ-P1-2-treated cells vs. vehicle-treated cells, ^#^*p* < 0.05 for venetoclax- or THZ-P1-2-treated cells *versus* combination treatment at the corresponding doses; ANOVA and Bonferroni post-test.

**Supplementary Figure 2. THZ-P1-2 induces cell differentiation in acute myeloid leukemia cellular models.** OCI-AML3, THP-1, and NB4 cells were exposed to vehicle or THZ-P1-2 (1.6, 3.2, and 6.4 μM) for 72 h. The histogram represents the mean fluorescence intensity (M.F.I.) for PE-CD11b **(A)** and APC-CD14 **(B)** expression. Bar graphs represent the mean ± SD of at least three independent experiments. The *p* values are indicated; **p* < 0.05, ***p* < 0.01, ****p* < 0.001; ANOVA and Bonferroni post-test. **(C)** Cytospin preparations were stained with Rosenfeld. The cell lines and THZ-P1-2 concentrations are indicated in the images. Scale bar = 50 μm. Black arrows indicate cells with a higher cytoplasm/nucleus ratio, red arrows indicate bent/folded nucleus, both features associated with a more differentiated phenotype in myeloid cells.
