# Supplementary material for: The PIP4K2 inhibitor THZ-P1-2 exhibits antileukemia activity by disruption of mitochondrial homeostasis and autophagy
Source: Blood Cancer J. 2022 Nov 9;12(11):151. doi: 10.1038/s41408-022-00747-w (PMC9643393; doi:10.1038/s41408-022-00747-w)
Supplement: Supplementary file 7 — Supplementary Table 4 [file 41408_2022_747_MOESM7_ESM.doc]

| **Supplementary Table 4.** Primer sequences and concentrations. | | |
| --- | --- | --- |
| **Gene** | **Sequence** | **Concentration** |
| *PIP4K2A* | FW: CCACCGTTTGTCTGTGTATAGGA  RV: TTCAGAGTTGGCAGTTCTTTGG | 300 nM |
| *PIP4K2B* | FW: TGCTGCCAAAACGGTGAAA  RV: CGTTGAAGCGTTTGGAGTACTG | 300 nM |
| *PIP4K2C* | FW: CCAGTGAGGACATTGCTGACAT  RV: CAGAAGCGTGTTGCCATGG | 300 nM |
| *ATG5* | FW: GGGCCATCAATCGGAAAC  RV: AGCCACAGGACGAAACAG | 300 nM |
| *ATG7* | FW: CGTTGCCCACAGCATCATCTTC  RV: TCCCATGCCTCCTTTCTGGTTC | 300 nM |
| *ATG10* | FW: TACGCAACAGGAACATCCA  RV: AACAACTGGCCCTACAATGC | 300 nM |
| *BAD* | FW: CACCAGCAGGAGCAGCCAAC  RV: CGACTCCGGATCTCCACAGC | 300 nM |
| *BAX* | FW: GAGCTGCAGAGGATGATTGC  RV: CAGCTGCCACTCGGAAAA | 300 nM |
| *BBC3* | FW: GACCTCAACGCACAGTACGAG  RV: AGGAGTCCCATGATGAGATTG | 300 nM |
| *BCL2* | FW: ATGTGTGTGGAGAGCGTCAA  RV: ACAGTTCCACAAAGGCATCC | 300 nM |
| *BCL2L11* | FW: ATGTCTGACTCTGACTCTCG  RV: CCTTGTGGCTCTGTCTGTAG | 300 nM |
| *BECN1* | FW: TCTGAAGAGGACCTGGACCCT  RV: GGCTCACGTCCATCTCGTC | 300 nM |
| *BNIP3* | FW: ATATGGGATTGGTCAAGTCGG  RV: CGCTCGTGTTCCTCATGCT | 300 nM |
| *CCNA2* | FW: GCCTTTCATTTAGCACTCTACA  RV: CAGGGTATATCCAGTCTTTCG | 300 nM |
| *CCNB1* | FW: GTCTCCATTATTGATCGGTTCATG  RV: CCAATTTCTGGAGGGTACATTTCT | 300 nM |
| *CCND1* | FW: CTCGGTGTCCTACTTCAAATG  RV: AGCGGTCCAGGTAGTTCAT | 300 nM |
| *CCNE1* | FW: TATATGGCGACACAAGAAAATG  RV: GTGCAACTTTGGAGGATAGA | 300 nM |
| *CDKN1A* | FW: TGTCACTGTCTTGTACCCTTGT  RV: GCCGGCGTTTGGAGTGGTAG | 300 nM |
| *CDKN1B* | FW: ACTCTGAGGACACGCATTTGGT  RV: TCTGTTCTGTTGGCTCTTTTGTT | 300 nM |
| *MCL1* | FW: GTAATAACACCAGTACGGACGG  RV: TCCCGAAGGTACCGAGAGAT | 300 nM |
| *MP1LC3B* | FW: AAGGCGCTTACAGCTCAATG  RV: CTGGGAGGCATAGACCATGT | 300 nM |
| *PMAIP1* | FW: CGCGCAAGAACGCTCAACC  RV: CACACTCGACTTCCAGCTCTGCT | 300 nM |
| *ULK1* | FW: CAGACAGCCTGATGTGCAGT  RV: CAGGGTGGGGATGGAGAT | 300 nM |
| *ACTB* | FW: AGGCCAACCGCGAGAAG  RV: ACAGCCTGGATAGCAACGTACA | 150 nM |
| *HPRT1* | FW: GAACGTCTTGCTCGAGATGTGA  RV: TCCAGCAGGTCAGCAAAGAAT | 150 nM |
