# Supplementary material for: The PIP4K2 inhibitor THZ-P1-2 exhibits antileukemia activity by disruption of mitochondrial homeostasis and autophagy
Source: Blood Cancer J. 2022 Nov 9;12(11):151. doi: 10.1038/s41408-022-00747-w (PMC9643393; doi:10.1038/s41408-022-00747-w)
Supplement: Supplementary file 8 — Supplementary Table 5 [file 41408_2022_747_MOESM8_ESM.doc]

| **Supplementary Table 5.** Cell cycle-, apoptosis-, and autophagy-related genes modulated by THZ-P1-2 in acute leukemia cells. | | | | | | | | | | | | |
| --- | --- | --- | --- | --- | --- | --- | --- | --- | --- | --- | --- | --- |
| **Gene** | **MV4-11 cells** | | | **OCI-AML3 cells** | | | **Jurkat cells** | | | **NALM6 cells** | | |
|  | **FC**^1^ | **S.D.** | ***p***^2^ | **FC**^1^ | **S.D.** | ***p***^2^ | **FC**^1^ | **S.D.** | ***p***^2^ | **FC**^1^ | **S.D.** | ***p***^2^ |
| *CCNA2* | 0.57 | 0.08 | **0.002** | 0.78 | 0.04 | **0.0016** | 1.06 | 0.49 | 0.8178 | 1.33 | 0.15 | **0.021** |
| *CCNB1* | 0.89 | 0.19 | **0.0009** | 0.89 | 0.10 | 0.1082 | 0.97 | 0.19 | 0.7479 | 1.22 | 0.02 | **0.0003** |
| *CCND1* | 0.31 | 0.05 | **0.0001** | 0.91 | 0.09 | 0.1346 | 4.33 | 1.71 | **0.0298** | n.d. | n.d. | > 0.05 |
| *CCNE1* | 0.96 | 0.12 | 0.5821 | 0.95 | 0.19 | 0.6258 | 1.29 | 0.10 | **0.0117** | 1.19 | 0.02 | **0.0004** |
| *CDKN1A* | 0.98 | 0.13 | 0.7495 | 4.48 | 0.42 | **0.0005** | 4.47 | 1.88 | **0.0345** | 0.59 | 0.09 | **0.0029** |
| *CDKN1B* | 2.43 | 0.70 | **0.0268** | 2.39 | 0.11 | **0.0001** | 2.45 | 0.16 | **0.0004** | 2.23 | 0.25 | **0.0023** |
| *BCL2* | 1.10 | 0.07 | 0.0626 | 0.87 | 0.09 | 0.1848 | 1.73 | 0.24 | **0.0088** | 0.44 | 0.02 | **< 0.0001** |
| *BCL2L1* | 1.68 | 0.15 | **0.0028** | 1.75 | 0.04 | **< 0.0001** | 1.38 | 0.34 | 0.1066 | 1.91 | 0.14 | **0.0009** |
| *MCL1* | 1.70 | 0.30 | **0.0186** | 1.80 | 0.26 | **0.009** | 1.85 | 0.33 | **0.0141** | 1.40 | 0.22 | **0.0391** |
| *BAX* | 1.36 | 0.13 | **0.0113** | 2.42 | 0.24 | **0.0013** | 1.60 | 0.11 | **0.0017** | 1.38 | 0.18 | **0.0235** |
| *BAD* | 1.33 | 0.21 | 0.0546 | 1.07 | 0.19 | 0.5175 | 2.39 | 1.04 | 0.0749 | 1.16 | 0.31 | 0.3674 |
| *BCL2L11* | 2.92 | 0.22 | **0.0004** | 2.00 | 0.08 | **0.0001** | 2.04 | 0.22 | **0.0025** | 1.66 | 0.14 | **0.0026** |
| *BBC3* | 1.89 | 0.30 | **0.0098** | 5.20 | 0.44 | **0.0003** | 5.75 | 2.19 | **0.0227** | 2.07 | 0.39 | **0.0123** |
| *PMAIP1* | 0.53 | 0.22 | **0.0125** | 1.11 | 0.18 | 0.3208 | 1.50 | 0.72 | 0.2572 | 1.51 | 0.20 | **0.0146** |
| *GADD45A* | 1.24 | 0.23 | 0.1095 | 3.29 | 0.13 | **< 0.0001** | 2.85 | 0.30 | **0.0011** | 1.57 | 0.20 | **0.0109** |
| *ULK1* | 1.74 | 0.17 | **0.003** | 2.69 | 0.16 | **0.0002** | 1.10 | 0.08 | 0.084 | 1.70 | 0.18 | **0.0047** |
| *MAP1LC3B* | 2.54 | 0.42 | **0.0051** | 4.43 | 0.22 | **< 0.0001** | 2.23 | 0.25 | **0.0023** | 1.88 | 0.27 | **0.0075** |
| *BECN1* | 1.83 | 0.34 | **0.016** | 1.34 | 0.20 | **0.0449** | 0.98 | 0.19 | 0.8227 | 1.31 | 0.23 | 0.0773 |
| *BNIP3* | 1.41 | 0.28 | 0.0638 | 1.54 | 0.16 | **0.0067** | 1.42 | 0.13 | **0.0075** | 1.41 | 0.20 | **0.0258** |
| *ATG5* | 1.70 | 0.24 | **0.0108** | 2.09 | 0.23 | **0.0025** | 2.08 | 0.57 | **0.0317** | 1.54 | 0.20 | **0.013** |
| *ATG7* | 1.63 | 0.17 | **0.0055** | 2.99 | 0.32 | **0.0011** | 0.61 | 0.04 | **0.0003** | 0.97 | 0.05 | 0.2926 |
| *ATG10* | 1.17 | 0.12 | 0.0688 | 1.12 | 0.16 | 0.2218 | 0.56 | 0.10 | **0.0031** | 1.07 | 0.24 | 0.6034 |

Abbreviation: F.C., Fold-change; S.D., standard deviation; n.d., not detected.

^1^Fold-change of vehicle-treated cells.

^2^Student *t* test.
