# Supplementary material for: The PIP4K2 inhibitor THZ-P1-2 exhibits antileukemia activity by disruption of mitochondrial homeostasis and autophagy
Source: Blood Cancer J. 2022 Nov 9;12(11):151. doi: 10.1038/s41408-022-00747-w (PMC9643393; doi:10.1038/s41408-022-00747-w)
Supplement: Supplementary file 9 — Supplementary Table 6 [file 41408_2022_747_MOESM9_ESM.doc]

| **Supplementary Table 6.** Association between *PIP4K2A*, *PIP4K2B*, and *PIP4K2C* mRNA levels and *ex vivo* response to THZ-P1-2 in leukemia. | | | |
| --- | --- | --- | --- |
| Cohort | Gene | *r* | *p** |
| AML – FM-USP | *PIP4K2A* | 0.10 | 0.72 |
|  | *PIP4K2B* | -0.28 | 0.31 |
|  | *PIP4K2C* | -0.15 | 0.59 |
| ALL - FM-USP | *PIP4K2A* | -0.25 | 0.29 |
|  | *PIP4K2B* | 0.13 | 0.59 |
|  | *PIP4K2C* | 0.26 | 0.28 |
| AML - UMCG | *PIP4K2A* | 0.12 | 0.61 |
|  | *PIP4K2B* | -0.32 | 0.14 |
|  | *PIP4K2C* | -0.07 | 0.76 |

*Spearman correlation test.
